# Systemic Infection with Enteric Adenovirus in Immunocompetent Child with *Haemophilus influenzae* Disease

**DOI:** 10.3201/eid1502.081066

**Published:** 2009-02

**Authors:** Nino Khetsuriani, Suxiang Tong, Xiaoyan Lu, Shannon Reed, Dean Erdman, Angela Campbell, Krongkaew Supawat, Sahas Liamsuwan, Narayanan Jothikumar, Sonja Olsen

**Affiliations:** Centers for Disease Control and Prevention, Atlanta, Georgia, USA (N. Khetsuriani, S. Tong, X. Lu, S. Reed, D. Erdman, A. Campbell, N. Jothikumar); National Institute of Health, Nonthaburi, Thailand (K. Supawat); Queen Sirikit National Institute of Child Health, Bangkok, Thailand (S. Liamsuwan); International Emerging Infections Program, Nonthaburi (S. Olsen); 1 Current affiliation: University of Washington, Seattle, Washington, USA.; 2 Current affiliation: Centers for Disease Control and Prevention, Atlanta, Georgia, USA.

**Keywords:** human adenoviruses, enteric adenoviruses, species F adenoviruses, adenovirus 40, encephalitis, meningitis, acute CNS illness, immunocompetent child, Thailand, letter

**To the Editor:** Recent articles have reported enteric human adenoviruses (HAdVs) types 40 and 41, previously thought to be restricted to the gastrointestinal tract (*1*), in multiple organ systems of a deceased immunodeficient child (*2*) and in respiratory specimens of children with acute respiratory illnesses (*3*). Here we present a case in which enteric HAdV-40 was found in the cerebrospinal fluid (CSF) and blood of an apparently immunocompetent child with *Haemophilus influenzae* invasive disease.

The patient, a 10-month-old previously healthy Thai boy, met the criteria for a clinical case of encephalitis (*4*) and, after informed consent was obtained, was enrolled in the study of causes of encephalitis in Thailand (collaboration between the US Centers for Disease Control and Prevention [CDC] and the Ministry of Health of Thailand). Clinical and laboratory information was collected from the medical record. Biologic specimens were sent to CDC and to the Thailand National Institute of Health, Nonthaburi, Thailand, for extensive testing for a broad range of pathogens potentially associated with encephalitis (*4*). Data on the clinical course of the patient are presented in the Table.

**Table:** Clinical course of illness in 10-month-old boy with systemic infection with enteric adenovirus and *Haemophilus influenzae* disease, Thailand, 2003–2004*

Date, 2003	Events
Dec 7	Patient hospitalized with 6-day history of fever >38°C and somnolence; blood culture positive for *Hemophilus influenzae*; isolate not typed (unavailable for further characterization)
Dec 9	CSF results: pleocytosis (2,710 leukocytes/mm^3^, 94% neutrophils); protein 178 mg/dL; glucose 11 mg/dL; CSF culture positive for *H. influenzae;* CSF Gram stain positive for gram-negative coccobacilli; antimicrobial drug treatment (ceftriaxone) started
Dec 11	New onset seizures, ataxia, and maculopapular rash on entire trunk and all extremities; no diarrhea or respiratory symptoms; brain ultrasound scan results within normal limits; anticonvulsant therapy (phenobarbital) started
Dec 12	CSF results: pleocytosis (100 leukocytes/mm^3^, 60% neutrophils, 40% monocytes); protein 131.6 mg/dL; glucose 31 mg/dL; CSF bacterial culture, results negative; CSF Gram stain results negative; patient enrolled in the encephalitis study; initial specimens for the study collected
Dec 22	Brain ultrasound scan results within normal limits; antimicrobial drug treatment (ceftriaxone) discontinued
Dec 23	CSF bacterial culture results negative; CSF Gram stain results negative; anticonvulsant therapy (phenobarbital) discontinued
Dec 27	Patient discharged in improved condition; discharge diagnosis: *H. influenzae* meningitis and septicemia
Jan 7†	Follow-up visit: full recovery without sequelae; convalescent-phase serum specimen obtained

*CSF, cerebrospinal fluid. †2004.

HAdV DNA was first detected in the CSF specimen collected on December 12, 2003, by an in-house pan-AdV PCR screening assay conducted as part of the study protocol. Amplicon sequences obtained closely matched that of HAdV-40. This unexpected result was confirmed by independent PCR assays on separate aliquots of the same specimen, a broadly reactive real-time TaqMan PCR, targeting the hexon gene and a HAdV 40/41 type-specific real-time Förster resonance energy transfer (FRET) PCR assay targeting the fiber gene (*5*). Sequences of the hexon gene hypervariable regions 1–6 that provide type specificity (*6*) showed a single nonsynonymous base substitution (C→T; Thr→Ile) at nucleotide position 107 of the HAdV-40 prototype strain Dugan (GenBank accession no. DQ115441).

HAdV-40 DNA with identical sequences was also detected in the acute-phase serum specimen also collected on December 12, 2003, but not in the convalescent-phase specimen collected on January 7, 2004. An oropharyngeal swab specimen obtained on December 12 was PCR-negative for HAdV DNA. Although no increase in levels of HAdV antibodies was detected by indirect enzyme immunoassay against pan-AdV antigen, microneutralization assay demonstrated a rise in levels of type-specific neutralizing antibodies to HAdV-40 between the acute-phase (<1:10) and convalescent-phase (1:40) serum specimens.

The results of other testing conducted on the same specimens as part of the study protocol were the following. CSF obtained on December 12, 2003, was negative by broad-specificity PCRs for bacterial 16S RNA and viral agents (alphaviruses, flaviviruses, bunyaviruses, human herpesviruses) as well as by PCR for enteroviruses, herpes simplex virus, Nipah virus, *Mycoplasma pneumoniae*, and *Neisseria meningitidis.* CSF was also negative for *Cryptococcus* spp. by India ink technique. Serum was negative for acute infection with flaviviruses (dengue and Japanese encephalitis viruses); alphaviruses (chikungunya virus); influenza viruses; human parainfluenza viruses 1–3; measles, mumps, and rubella viruses; enteroviruses; *Bartonella henselae;* rickettsiae (*R. typhi, Orientia tsutsugamushi,* and *R. conorii*); and *M. pneumoniae.* Results of PCR on saliva specimens and serologic testing for rabies were negative; an oropharyngeal swab specimen was negative by PCR for *M. pneumoniae*; and results of a smear for malaria parasites were negative. The patient was HIV negative.

Detection of HAdV-40 in CSF in this case was confirmed by multiple PCRs with amplicon sequencing. Detection of virus in the acute-phase serum specimen confirms systemic infection and demonstrates that HAdV-40 DNA found in CSF did not arise from contamination of the CSF at the time of collection. Laboratory contamination is also unlikely because the nucleotide sequence of the identified strain (GenBank accession no. FJ228470) was not identical to the prototype reference strain used for positive control in the PCR. Seroconversion to HAdV-40 provides further evidence that this child experienced an acute systemic infection with this virus.

The contribution of HAdV-40 to the clinical illness in this patient remains unclear. He had a confirmed *H. influenzae* invasive infection, which likely explains the initial underlying illness. However, the detection of HAdV-40 coincided in time with the development of neurologic signs (new-onset seizures, ataxia) and widespread rash. By then, the patient had been receiving antimicrobial drug therapy for several days, his CSF was negative for 16S bacterial RNA by PCR and culture-negative for *H. influenzae*, and the CSF pleocytosis had decreased substantially. These circumstances make it less likely that these signs were associated with the underlying *H. influenzae* disease and raise the possibility that superimposed HAdV-40 infection played a role. Because the patient had no diarrhea or respiratory symptoms, no evidence of immunodeficiency, no stool specimen available for testing, and no evidence of HAdV in throat swab specimen, the pathogenesis of HAdV-40 infection in this case is unknown. The origin of the maculopapular rash concurrent with neurologic symptoms in this patient is also unclear. Rash is not typical for *H. influenzae* infection and, although reported for some HAdV infections (*7*), has not been previously described for HAdV-40/41.

In conclusion, this case demonstrates the possibility of nongastroenteric, systemic infection involving CNS with enteric HAdV in immunocompetent hosts. Broad-specificity AdV PCR assay followed by amplicon sequencing enabled detection of this pathogen in an unexpected context and can be useful in defining the nongastroenteric disease effects associated with the enteric HAdVs.
